# Risk factors associated with extended spectrum beta lactamase *Klebsiella pneum*oniae outbreak in a neonatal intensive care unit

**DOI:** 10.1017/ash.2026.10785

**Published:** 2026-07-10

**Authors:** Areej Yousef Al Ali, Gautam Hebbar, Dalal Al-Ghanim, Khaled Alkulaib, Nasser Althefiri, Kholoud Al-Fadhalah

**Affiliations:** 1 Infection Control Directorate, Kuwait Ministry of Health, Kuwait; 2 Kuwait University Faculty of Medicine, Kuwait

## Abstract

**Objective:**

(1) identify risk factors for extended-spectrum beta-lactamase *Klebsiella pneumoniae* (ESBL-KP) acquisition in the neonatal intensive care unit (NICU) and (2) evaluate the clinical impact of ESBL-KP acquisition on neonatal outcomes at NICU discharge.

**Design:**

This retrospective case–control study (Aug 2022–Sept 2025) compared neonates who acquired ESBL-KP in the NICU (cases) with those who did not (controls).

**Patients:**

600 neonates admitted to a Kuwaiti NICU located in a general hospital.

**Methods:**

Data included clinical, demographic, antibiotic, and laboratory records. The primary outcome was ESBL-KP acquisition. The secondary outcome was status at NICU discharge. Regression analysis was used to evaluate associations for the primary and secondary outcomes.

**Results:**

Of 600 neonates, 30% acquired ESBL-KP. Among these, 12% had bloodstream infections (BSIs). Length of NICU stay (OR:1.02, 95% CI:1.01–1.03), intrauterine growth restriction (IUGR) (OR:2.59, 95% CI:1.08–6.24), extreme-prematurity (OR:2.89, 95% CI:1.29–6.47), previous hospital admission (OR:4.45, 95% CI:1.75–11.32), and prior ampicillin use (OR:3.51, 95% CI:2.12–5.82) were statistically significant risk factors for acquisition of ESBL-KP in the adjusted regression model. Moreover, ESBL-KP-positive neonates faced 12.83 times greater odds of death from sepsis (95 % CI: 2.41–68.22), than ESBL-KP negative neonates.

**Conclusions:**

ESBL-KP acquisition was strongly linked to IUGR, extreme prematurity, previous admission, and ampicillin use. While a longer hospital stay correlated with acquisition, this relationship is prone to time-dependent bias and reverse causation. Acquisition also raised sepsis-related mortality risk, though small sample sizes require cautious interpretation. Targeted prevention is essential to improve neonatal outcomes.

## Introduction

Extended spectrum beta-lactamase producing *Klebsiella pneumoniae* (ESBL-KP) have been an escalating public health concern around the world.^1^ The US Centers for Disease Control and Prevention has labeled it as a “serious threat” and the World Health Organization has designated it as a critical priority for antibiotic development.^2,3^

In Kuwait, healthcare associated infections (HAIs) surveillance data from 2016 to 2024, revealed an increase in ESBL-KP acquisition in the neonatal intensive care units (NICUs). Latest data from 2024 showed that ESBL-KP made up around 15% of HAIs in the NICUs. Likewise, 15.6% of all primary bloodstream infections (BSIs) acquired in the NICUs were caused by ESBL-KP, thus highlighting the growing concern regarding ESBL-KP in NICUs across Kuwait

ESBL-KP has been a noted cause of outbreaks in NICUs, with an increasing number of reports highlighting their association with high morbidity and mortality rates.^4–6^ The two main factors facilitating the outbreaks of ESBL-KP are: (1) horizontal transmission where spread occurs primarily through healthcare workers’ hands, contaminated reusable devices and environmental surfaces and (2) inappropriate use of antibiotics.^4,7^

The unique characteristics and patient population of the NICU presents specific challenges to infection control implementation. Although the conventional pattern of use in the NICU is the ratio of nurse per patient equal to 1:1, the increased ratio of nurses to patients in the NICU can hinder infection control practices, thus affecting infection cross-contamination^8^ Overcrowding is also common in the NICU as the need for specialized care is frequently greater than the amount of space available, which can lead to a violation of standard infection prevention protocols.^9^ We find several neonatal risk factors for HAIs from the literature that includes prolonged use of antibiotics, low gestational age, and long hospital stay.^4,10^ These studies were mostly small, limited to utilization of antibiotics, and did not systematically examine risk factors nor control for them using multivariable regression analysis.^11^ These methodology limitations emphasize that further larger studies employing stronger statistical methods to elucidate the factors that play a role in infection among this population are needed. Insight into these determinants is critical to develop effective screening, diagnosis, and early prevention efforts. We want to profile and quantify the risk factors associated with ESBL-KP acquisition in these neonates and estimate the clinical effect of ESBL-KP acquisition on the neonatal outcomes upon NICU discharge.

## Method

### Study design

This retrospective case-control study investigated ESBL-KP acquisition in a 51-incubator, level III NICU in Kuwait (July 2022–September 2025). Cases were neonates with a positive ESBL-KP culture (clinical or surveillance) after three days of admission following a prior negative result. Controls were non-case neonates admitted during the same period, selected at a 3:1 ratio. Neonates with missing records, unprocessed cultures, or who remained hospitalized at dataset lock were excluded.

### Active screening methods

During the study period, rectal swabs were obtained from all neonates admitted to the NICU, in addition to weekly screening until NICU discharge or death.

### Data collection

Data on demographic (eg, gestational age, birth weight, sex), clinical (eg, history of admission diagnosis, history of HAI), laboratory (eg, history of other multidrug-resistant organism-MDRO), antibiotic prescriptions, were collected (Supplemental Table 1).

### Outcome variables

The primary outcome was ESBL-KP acquisition, recorded as a binary variable on cultures obtained during the NICU admission, indicated as either infection or colonization, according to the case definition. Infection required a positive clinical culture with associated symptoms, whereas colonization was defined as positive clinical or surveillance cultures without clinical symptoms.

The secondary outcome was NICU discharge status, categorized as: (1) survival, (2) mortality due to sepsis, or (3) mortality due to other causes. Mortality was defined by official death certificate documentation.

### Data management and analysis

STATA 16.1 was used to analyze data, with a *P* value of less than .05, considered statistically significant.

To prevent multicollinearity, birth weight was excluded from the regression analysis due to high correlation with gestational age (*r* = .86, *P* value <.001). For the same reason, “history of HAI” was restricted to descriptive analysis, as it overlapped heavily with ESBL-KP acquisition.

Due to the very small number of patients who received metronidazole or levofloxacin, these two antibiotic variables were excluded from the regression analysis. In addition, the “prematurity” variable was omitted from the multivariable multinomial analysis as the “very-preterm” category had zero cells for the death due to sepsis outcome.

A sensitivity analysis was conducted to explore whether risk factors differed between neonates with ESBL-KP colonization and those with ESBL-KP bloodstream infection (BSI) using a categorical outcome (controls, colonization only, BSI). Due to the small sample size in the BSI group, this model displayed a severe loss of statistical power, resulting in less precise estimates and wide 95% confidence intervals (CIs). Furthermore, risk variables did not distinctly differ between the colonized and infected groups versus the control group. Therefore, combining them into a single binary outcome (ESBL-KP acquisition: Yes vs No) was the most methodologically sound approach.

### Statistical methods used for analysis

Study population characteristics were described using frequencies and percentages for categorical variables, and means or medians for continuous variables, as appropriate.

With regards to the primary outcome, *χ* ^2^ analysis was used to evaluate the distribution of exploratory variables across ESBL-KP positive cases only, stratified by sample type (blood vs colonization) using a variable created within the ESBL-KP positive cohort (1 = rectal colonization, 2 = BSI). Additionally, univariable and multivariable logistic regression analyses were used to examine the independent associations between ESBL-KP acquisition and risk factors.

With regards to the secondary outcome, *χ* ^2^ test and multinomial logistic regression analyses were utilized to investigate the relationship between ESBL-KP acquisition and neonatal status at NICU discharge.

All variables included in the univariable analysis were based on clinically driven factors derived from the literature. Only covariates that were significantly associated with the outcome of interest in the univariable model were included in the final adjusted multivariable regression model. The “history of MDRO isolation (other than ESBL-KP)” and “cefotaxime” variables were included in the final adjusted model as a priori selection regardless of the significance level in the univariable analysis.

## Results

### Sample characteristics

The final analysis included 600 neonates: 177 cases and 423 controls. Boys comprised 60% of cases (n = 106) and 55% of controls (n = 233) (Table 1). Compared to controls, cases had a lower mean birth weight (1,436 g vs 2,143 g) and a lower mean gestational age (29.95 vs 33.37 wk) (Table 2). Conversely, cases had a longer mean length of stay (LOS) (59.58 vs 29.70 d) (Table 2). Among cases, the mean time from admission to ESBL-KP acquisition was 33 days (data not shown).

**Table 1.** Descriptive analysis of the neonates enrolled in the case-control study (n = 600)

Variable	Cases, n (%)	Control, n (%)
Gender		
Male	106 (60)	233 (55)
Female	71 (40)	190 (45)
Admission status		
New admission	161 (91)	409 (97)
History of readmission/transfer	16 (9)	14 (3)
Room		
R1	65 (37)	130 (31)
R2	45 (25)	119 (28)
R3	51 (29)	133 (31)
R4	16 (9)	41 (10)
Prematurity		
Full term (≥37 weeks)	16 (9)	150 (35)
Moderate-late preterm (32–36 weeks)	40 (23)	125 (30)
Very-preterm (28–<32 weeks)	67 (38)	91 (22)
Extremely preterm (<28 weeks)	54 (30)	57 (13)
Mode of delivery		
SVD	52 (29)	161 (38)
Elective C-section	3 (2)	15 (4)
Emergency C-section	122 (69)	247 (58)
Neonatal status at NICU discharge		
Survived	164 (93)	384 (91)
Died-Sepsis	11 (6)	2 (<1)
Died-other	2 (1)	37 (9)
Diagnosis on admission		
Others	24 (13)	124 (29)
RDS	134 (76)	273 (65)
IUGR	19 (11)	26 (6)
MDRO-Other than ESBL		
No	159 (90)	402 (95)
Yes	18 (10)	21 (5)
HAI		
No	139 (79)	362 (86)
Yes	38 (21)	61 (14)

**Table 2.** Descriptive analysis of continuous variables among neonates enrolled in the case control study (n = 600)

	Mean (S.D)		Median (IQR)		Min - max	
Variable	Cases	Controls	Cases	Controls	Cases	Controls
GA - weeks	29.95 (4.11)	33.37 (4.84)	29.00 (33–27)	34 (37–30)	23–41	22–41
BW- grams	1,436 (758.60)	2,143.12 (997.10)	1,180 (1700–940)	2,100 (2980–1,300)	520–4,040	510–5,270
LOS - days	59.58 (39.71)	29.70 (35.26)	55 (78–33)	16 (40–9)	6–235	0–382

GA: Gestational age, BW: Birth weight, LOS: Length of stay.

Respiratory distress syndrome (RDS) was the most common admission diagnosis for both cases (76%) and controls (65%) (Table 1). Only 9% of cases were full-term, compared to 35% of controls (Table 1). Nearly all neonates received antibiotic therapy (99% of cases vs 98% of controls), most frequently gentamicin (76% vs 77%) and ampicillin (83% vs 58%) (Table 3).

**Table 3.** Descriptive analysis of antibiotics prescribed among the neonates enrolled in the case control study (n = 600)

Variable	Cases, n (%)	Controls, n (%)
Overall antibiotics used		
No	1 (1)	10 (2)
Yes	176 (99)	413 (98)
Ampicillin		
No	30 (17)	176 (42)
Yes	147 (83)	247 (58)
Gentamicin		
No	42 (24)	99 (23)
Yes	135 (76)	324 (77)
Amikacin		
No	111 (63)	355 (84)
Yes	66 (37)	68 (16)
Metronidazole		
No	168 (95)	419 (99)
Yes	9 (5)	4 (1)
Cefotaxime		
No	146 (83)	364 (86)
Yes	31(17)	59 (14)
Meropenem/imipenem		
No	133 (75)	363 (86)
Yes	44 (25)	60 (14)
Vancomycin		
No	164 (93)	406 (96)
Yes	13 (7)	17 (4)
Piperacillin/tazobactam		
No	112 (63)	244 (58)
Yes	65(37)	179 (42)
Teicoplanin		
No	147 (83)	406 (96)
Yes	30 (17)	17 (4)
Levofloxacin		
No	166 (94)	422 (100)
Yes	11 (6)	1 (<1)

Approximately, two-thirds of the neonates were delivered via C-section, the majority were emergency C-section (69% for cases vs 58% for controls) (Table 1). Compared to controls, cases had a higher prevalence of previous readmission or transfer (9% vs 3%) and a history of other MDRO isolates (10% vs 5%) (Table 1).

Hospital-acquired infections (HAIs) occurred in 16.5% of the neonates (99/600), totaling 137 separate infections. Three-quarter of these were BSIs (Supplemental Figure 1). From these HAIs, 130 pathogens were isolated, 65% of which were gram-negative (Supplemental Figure 2). Nearly 90% of these gram-negative bacteria were *Enterobacterales*, with *Klebsiella pneumoniae* comprising the majority (44/75) (data not shown).

### Primary outcome: ESBL-KP status

Almost one third of neonates (n = 177, 30%) had ESBL-KP detected (Table 1). The majority of the ESBL-KP isolates were obtained from rectal swabs (87%), while 13% were recovered from blood cultures (n = 22) (Supplemental Figure 3). Among the blood isolates, 19 were diagnosed as primary BSIs, of which 4 were central line-associated bloodstream infections (CLABSI). On the other hand, only 3 blood isolates were diagnosed as secondary BSIs, attributed to pneumonia and necrotizing enterocolitis (NEC).

### χ^2^ analysis of association between ESBL-KP positive cases and several variables

Comparing neonates with ESBL-KP positive BSI with ESBL-KP positive rectal colonization, no statistically significant differences were found between the two groups for most of the baseline demographics, comorbidities, or exposure variables including gender, mode of delivery, admission diagnosis, MDROs other than ESBL, admission status and history of use of ampicillin, gentamycin, tazocin, and cefotaxime. The only exceptions were specific prior antibiotic exposures (ie, amikacin, meropenem, teicoplanin, vancomycin), which were more frequently observed in the BSI group. In addition, with regards to the distribution of prematurity, the combined proportion of very preterm and extremely preterm infants was significantly higher in the BSI group than in the colonization group (82% vs 66%, *P* = .005) (Supplemental Table 5).

### Logistic regression analysis associating ESBL-KP acquisition with several variables

Multivariable logistic regression analysis (Table 4) indicated that after adjusting for different potential risk factors, the following variables remained significantly associated with higher odds of ESBL-KP acquisition: prematurity, admission diagnosis, LOS, history of readmission or transfer from another hospital, and use of ampicillin.

**Table 4.** Univariable and multivariable logistic regression analysis associating ESBL-KP acquisition with several risk factors (n = 600)

Covariate	Unadjusted OR (95% CI)	Adjusted OR (95%CI) #
Gender		
Male	Ref	
Female	0.81 (0.56–1.15)	–
Room number		
1	Ref	
2	0.76 (0.48–1.20)	–
3	0.76 (0.49–1.18)	–
4	0.74 (0.39–1.43)	–
Prematurity		
Term	Ref	Ref
Moderate-late-term	3.00 (1.60–5.61)**	1.96 (0.97–3.98)
Very-preterm (28 week–<32 weeks)	6.90 (3.77–12.63)***	3.66 (1.75–7.66)**
Ex-preterm (< 28 weeks)	8.88 (4.70–16.77)***	2.89 (1.29–6.47)*
Mode of delivery		
SVD	Ref	Ref
Elective C-section	0.62 (0.17–2.22)	0.68 (0.15–3.03)
Emergency C-section	1.52 (1.04–2.23)*	1.49 (0.96–2.33)
Admission diagnosis		
Other	Ref	Ref
RDS	2.53 (1.56–4.11)***	1.73 (0.93–3.21)
IUGR	3.77 (1.80–7.87)***	2.59 (1.08–6.24)*
MDROs—Other than ESBL-KP		
No	Ref	Ref
Yes	2.92 (1.12–4.17)*	1.08 (0.49–2.40)
Admission status		
New admission	Ref	Ref
History of readmission/transfer	2.90 (1.38–6.08)**	4.45 (1.75–11.32)**
LOS		
	1.03 (1.02–1.04)***	1.02 (1.01–1.03)**
Antibiotics–Ampicillin		
No	Ref	Ref
Yes	3.49 (2.25–5.40)***	3.51 (2.12–5.82)***
Antibiotics–Amikacin		
No	Ref	Ref
Yes	3.10 (2.08–4.63)***	1.80 (1.03–3.13)*
Use of antibiotics–Gentamycin		
No	Ref	
Yes	0.98 (0.64–1.48)	–
Use of antibiotics–Tazocin		
No	Ref	
Yes	0.79 (0.55–1.14)	–
Use of antibiotics–Cefotaxime		
No	Ref	
Yes	1.31 (0.81–2.10)	1.47 (0.82–2.64)
Use of antibiotics–Meropenem		
No	Ref	
Yes	2.00 (1.29–3.10)**	0.70 (0.35–1.40)
Use of antibiotics–Teicoplanin		
No	Ref	Ref
Yes	4.87 (2.61–9.09)***	2.28 (1.04–5.00)*
Use of antibiotics–Vancomycin		
No	Ref	
Yes	1.89 (0.90–3.98)	–

*P value <.05.**P value <.01.***P value <.001.

# Only covariates that were significantly associated with the outcome of interest in the univariable model were included in the multivariable regression except for variables selected a priori.

In the multivariable logistic regression model (Table 4), very-preterm neonates had more than three times the odds of ESBL-KP acquisition (OR = 3.66, 95% CI: 1.75–7.66), while extremely-preterm had 2.89 the odds (OR = 2.89, 95% CI: 1.29–6.47) compared to full-term neonates. Neonates diagnosed with IUGR had 2.59 the odds of ESBL-KP acquisition compared to those with other diagnoses (95% CI: 1.08–6.24). History of readmission was also associated with a more than fourfold increase in odds of ESBL-KP acquisition (OR = 4.45, 95% CI: 1.75–11.32). Furthermore, for each additional day of NICU stay, odds of ESBL-KP acquisition increase by 2% (OR: 1.02, 95% CI: 1.01–1.03).

Regarding antibiotics use, ampicillin use was associated with a 3.51—fold increase in odds of ESBL-KP acquisition (95% CI: 2.12–5.82) compared to neonates who didn’t use this antibiotic. Although amikacin and teicoplanin use were found to be highly significant in the univariable analysis (*P* < .001), in the multivariable model, after adjusting for risk factors and other confounders, the association between amikacin and teicoplanin with the primary outcome attenuated showing only borderline significance (*P* = .04 and *P* = .04, respectively).

### Secondary outcome: Neonatal status at NICU discharge

Most neonates in both cases and controls survived to discharge. Among cases, 13 (7%) of the neonates died, out of which the majority of deaths were attributed to sepsis (11 out of the 13 neonates). On the contrary, among controls, 39 (10%) of the neonates died, out of which the cause of death for almost all controls was other than sepsis (37 out of 39) (Table 1).

### χ^2^ analysis of association between ESBL-KP acquisition and neonatal status at NICU discharge

The prevalence of neonates that died due to sepsis in NICU was significantly higher among neonates with ESBL-KP positive (85%) than those with ESBL-KP negative (15%) (*P* < .001) (Table 5). Information about the ESBL-KP isolated by source/type is available in Supplemental Table 2.

**Table 5.** χ^2^ analysis of association between ESBL-KP acquisition (no vs yes) and neonatal status at NICU discharge (survive, died -sepsis, died-other causes) in a case-control study (n = 600)

		Prevalence n (%)			
Categorical variables	Total in category	Survived	Died-sepsis	Died-other	*P*-value
ESBL-KP					**<.001**
No	423	384 (70)	2 (15)	37 (95)	
Yes	177	164 (30)	11 (85)	2 (5)	

### Multinomial logistic regression analysis associating ESBL-KP acquisition with neonatal status at NICU discharge

To examine the association between ESBL-KP acquisition and neonatal status at NICU discharge, multinomial logistic regression model was applied (Supplemental Tables 3 and 4).

Using survival as the reference category (Supplemental Table 4), neonates who acquired ESBL-KP had more than tenfold higher risk of death due to sepsis compared to neonates without ESBL-KP in the fully adjusted model (RRR = 12.83, 95% CI: 2.41–68.22).

## Discussion

Prematurity was found to be significantly associated with the acquisition of ESBL-KP in this study. Very-preterm (OR = 3.66, 95% CI: 1.75–7.66) and extreme-preterm neonates (OR = 2.89, 95% CI: 1.29–6.47) had approximately three times higher odds of ESBL-KP acquisition compared to the full-term neonates in the fully adjusted model. These finding align with Rettedal et al study who reported preterm neonates having 7.6 times higher odds of ESBL-KP acquisition (95 % CI: 2.8–20.9) even after adjusting for other risk factors.^12^ Additionally, a large retrospective study in South Africa, involving 2,437 neonates, found that each additional week of gestation reduced the odds of multi-drug resistant *Enterobacteriaceae* (MDRE) infection by approximately 12% (OR = .881, 95% CI: .794–.977).^13^ Prematurity is a biological risk factor for infection due to immature immune systems and delicate skin barriers, which facilitate colonization.^6,14^

The present study found that, consistent with previous research,^15,16^ each additional day spent in the NICU increased the odds of ESBL-KP acquisition by 2% (95% CI: 1.01–1.03) in the fully adjusted model. However, this association warrants cautious interpretation due to inherent time-dependent bias; an extended hospital stay reflects a prolonged window of exposure to invasive devices, environmental reservoirs, and colonization pressure, rather than an independent baseline risk factor. Alternatively, reverse causation may play a role, as ESBL-KP acquisition itself can extend a neonate’s LOS relative to controls. To address these confounding factors, future studies should employ survival analyses, such as Cox proportional hazards modeling, to effectively decouple time-at-risk from clinical risk factors.

A history of readmission or transfer was another significant predictor of ESBL-KP acquisition, with affected neonates having more than four times the odds of acquisition of ESBL-KP compared to those without such history (OR = 4.45, 95% CI: 1.75–11.32) in the adjusted model. This aligns with a Moroccan study where transferred neonates from other wards or institutions had similarly increased odds of MDRE, predominantly ESBL-KP acquisition.^17^ Readmitted or transferred neonates act as reservoirs for transmission; therefore, routine admission screening and isolation are vital for prevention.

IUGR was also found to be a strong independent risk factor for ESBL-KP acquisition (OR = 2.59, 95% CI: 1.08–6.24), in the current study, after controlling for other risk factors. This is likely attributed to chronic placental insufficiency impairing fetal growth and weakened immune defences.^18–20^ IUGR neonates often experience respiratory issues like respiratory distress syndrome (RDS) and bronchopulmonary dysplasia (BPD), leading to prolonged need for mechanical ventilation, which is a well-established risk factor for ESBL acquisition.^19,20^

Our multivariable model found that only ampicillin (OR = 3.51, 95% CI: 2.12–5.82) remained a highly statistically significant risk factor associated with ESBL-KP acquisition. Ampicillin is one of the most commonly prescribed empiric antibiotics in the NICU and has been linked to increased ESBL-KP risk.^21,22^ The current study finding is concerning as widespread ampicillin use may bring significant selective pressure on resistant strains and cause infection or colonization with ESBL-producing pathogens.^11^ It must be noted that neonates who receive ampicillin often have a higher baseline severity of illness upon admission, which inherently increases their exposure to the healthcare environment and subsequent risk of MDRO acquisition. Therefore, the association with ampicillin use may reflect confounding by indication or illness severity rather than a direct causal effect.

In contrast to prior reports,^11,23,24^ multivariate adjustment eliminated the statistical significance of meropenem. Furthermore, amikacin and teicoplanin attenuated from strong univariate predictors to only borderline significant risk factors for ESBL-KP. This marginal significance suggests that while these antibiotics remain relevant, their independent contribution is modest once baseline risk is factored in.

In contrast to prior reports, vancomycin and third-generation cephalosporins were not independently associated with ESBL-KP acquisition.^11,25,26^ This suggests previous findings were likely confounded by disease severity rather than reflecting true independent risks, highlighting the critical role of multivariable analysis in controlling for such confounding.

This study also evaluated the impact of ESBL-KP acquisition on neonatal discharge outcomes. Multinomial regression revealed that ESBL-KP-positive neonates faced a nearly thirteen times higher risk of sepsis-related death compared to non-carriers (95% CI: 2.41–68.22). This lethality is linked to genetic markers like the rmpA gene, which facilitates a hypervirulent, immune-evading phenotype.^27^

This study’s case-control design was one of its major strengths which allowed for a direct comparison between neonates who acquired ESBL-KP and those who did not, despite having similar exposures. This approach enhanced the ability to identify factors associated with ESBL-KP acquisition. Additionally, the study’s large sample size, compared to several previous studies, enhanced its statistical power, thus increasing the reliability of the findings. The study was further strengthened by the inclusion of multiple risk factors in the multivariable regression models that allowed for identification of the independent effect of each factor while controlling for potential confounders.

However, several limitations apply. First, the single-center design limits external generalizability. Second, despite multivariable analysis, residual confounders such as invasive device use, colonization pressure, nurse-to-patient ratio, and cumulative antibiotic exposure may remain. Due to the retrospective nature of record extraction, detailed data for some of these parameters were unavailable. Third, treating NICU LOS as a static variable introduces time-dependent bias and potential for reverse causation, as ESBL-KP acquisition itself can extend hospitalization relative to controls. Future multicenter prospective studies utilizing Cox proportional hazards modeling are warranted to decouple time-at-risk from clinical factors. Additionally, the study lacks genotyping for virulence factors, and the retrospective design may introduce potential data inaccuracies. Finally, the small sample size of blood isolates limited our ability to fully differentiate predictors for systemic infection versus rectal colonization, resulting in wide 95% CIs. While combining colonization and BSI into a composite outcome ensured sufficient power for robust multivariable modeling, it may mask subtle differences unique to systemic invasion. Our sensitivity multivariable stratified analyses revealed no major differences but were underpowered given the few definitive BSIs. Similarly, because the study was powered for ESBL-KP acquisition rather than mortality, the low prevalence of neonatal deaths resulted in small subgroups and wide 95% CIs, advising future larger studies to firmly validate the effect size. Consequently, supplemental findings should be interpreted with caution.

In conclusion, the results of this study highlight the critical importance of prompt implementation of infection control interventions that specially target vulnerable neonates in the NICU. Encouraging careful antibiotic use and ensuring consistent application of clinical care standards will supplement these efforts. Although our data linked ESBL-KP acquisition to an increased risk of sepsis-related mortality, the small sample size in this subgroup necessitates a cautious interpretation.

## Supporting information

10.1017/ash.2026.10785.sm001 Al Ali et al. supplementary material Al Ali et al. supplementary material

## Supplementary material

The supplementary material for this article can be found at https://doi.org/10.1017/ash.2026.10785.
