# Supplementary material for: Risk factors associated with extended spectrum beta lactamase Klebsiella pneumoniae outbreak in a neonatal intensive care unit
Source: Antimicrob Steward Healthc Epidemiol. 2026 Jul 10;6(1):e210. doi: 10.1017/ash.2026.10785 (PMC13372728; doi:10.1017/ash.2026.10785)

**Supplementary Materials**

Supplemental Table 1: Description of variables included in the analysis

| **Variable name** | **Description/definitions** |
| --- | --- |
| Sex | A binary variable of Male/ Female was being used in the analysis. |
| Gestational age (GA) | Gestation age variable was collected from medical records as a continuous variable. |
| Prematurity | Pre-term is defined as babies born alive before 37 weeks of pregnancy are completed. Using GA variable, prematurity variable was generated and classified into four groups based on the WHO classification: full-term (born between 37 and 42 weeks of gestation), moderate to late pre-term (born between 32 and under 37 weeks of gestation), very pre-term (born between 28 and under 32 weeks of gestation) and extremely pre-term (born at less than 28 weeks of gestation). For both descriptive and covariate adjustment, prematurity variable was used as a categorical variable. |
| Birth weight (BW) | Birth weight was recorded in grams and used as a continuous variable. |
| Length of stay (LOS) | This variable, which was used as a continuous variable (days), was generated by determining the difference between the NICU admission date and the NICU date of discharge or in-unit death. LOS was the number of days between the date of admission to the NICU and the date of discharge or death from the unit. |
| Time to positivity | Defined as the number of days from neonatal admission to the first positive ESBL-KP culture, was calculated and analysed as a continuous variable in the descriptive analysis. |
| Admission diagnosis | The original admission diagnosis, as documented by NICU physician in the medical files, were recategorized into three categories for analysis: "Respiratory Distress Syndrome (RDS)", "Intrauterine Growth Restriction (IUGR)", and “Others". The “Others” category was created to group all remaining diagnosis due to their low frequency. |
| Admission status | Admission status was categorized into two groups: (1) new admission - first time admission to the NICU-, and (2) history of readmission/ transfer - which includes infants who were readmitted after being discharge to the ward, or those transferred back to the NICU from a tertiary hospital following specialized care (e.g., cardiac or other pediatric surgery). |
| Admission room | Since the NICU was comprised of four rooms, a categorical variable with four groups (Room1, Room2, Room3, and Room4) was collected and used in the analysis. |
| Mode of delivery | A categorical variable defining three groups - spontaneous vaginal delivery (SVD), elective cesarean delivery, and emergency cesarean delivery - was used in the analysis. |
| History of MDRO isolation (other than ESBL- KP) | Data regarding Multidrug Resistance Organism (MDRO) isolation history (both colonization and infection) were also obtained from the Laboratory Information System (LIS). This variable was categorized into two groups: (1) Yes – the neonate had any MDRO isolate other than of ESBL-KP, and (2) No- the neonate had no MDRO isolates other than ESBL-KP. |
| Prescribed antibiotics | A variable was generated to capture prescribed antibiotics in the NICU, encompassing ten different antibiotics: ampicillin, gentamicin, amikacin, metronidazole, cefotaxime, meropenem, vancomycin, teicoplanin, levofloxacin, and piperacillin-tazobactam. A combined binary variable indicating the presence or absence of any antibiotic prescription (Yes/No) was created and used in the descriptive analyses. Additionally, each of the above-mentioned antibiotics was analyzed separately as a binary variable (Yes/No) to assess the individual effect of each drug in the covariate adjustments. |
| HAIs | All patient-related healthcare-associated infections (HAIs) were also obtained, including those attributed to ESBL-KP. Using the U.S. Center for Disease Control and Prevention (CDC) definitions, HAIs were recoded as a binary variable (Yes/ No) and utilized in both the descriptive analysis and covariate adjustment. Additionally, a variable categorizing HAI type, was generated and used for descriptive analysis, grouping HAIs into five groups: "Primary Bloodstream Infection (BSI)", "Pneumonia", “Eye, Ear, Nose, Throat, and Mouth (ENT-CONJJ)”, “Necrotizing enterocolitis (NEC)”, and “Others". The “Others” category included all remaining diagnosis with low frequency. |

Supplemental Table 2**:** Chi-square analysis of association between ESBL-KP acquisition (no vs. yes) and neonatal status at NICU discharge (survive, died -sepsis, died-other causes) in a case-control study (n=600), stratified by sample source.

| **Categorical variables** | **Total in category** | **Prevalence**  **n (%)** | | | ***p*-value** |
| --- | --- | --- | --- | --- | --- |
|  |  | Survived | Died- sepsis | Died-other |  |
| ESBL-KP |  |  |  |  | **<0.001** |
| No | 423 | 384 (70) | 2 (15) | 37 (95) |  |
| Yes - rectal | 155 | 153 (28) | 1 (8) | 1 (2) |  |
| Yes - blood | 22 | 11 (2) | 10 (77) | 1 (3) |  |

Supplemental Table 3**:** Univariable multinomial logistic regression analysis associating outcome with several risk factors (n=600). The reference group is neonates who survived till NICU discharge.

|  | ***Died- sepsis*** | ***Died -other*** | ***Overall p-value*** |
| --- | --- | --- | --- |
| **Covariate** | **RRR (95%CI)** | **RRR (95%CI)** |  |
| ESBL |  |  | <0.001 |
| No | Ref | Ref |  |
| Yes | 12.87 *(2.82 - 58.47) | 0.12* (0.03-0.53) |  |
| LOS |  |  | <0.001 |
|  | 1.00 (0.99- 1.01) | 0.96*** (0.94-0.98) |  |
| Gender |  |  | 0.405 |
| Male | Ref | Ref |  |
| Female | 0.58 (0.17 – 1.91) | 1.38 (0.72-2.64) |  |
| Admission status |  |  | 0.218 |
| New admission | Ref | Ref |  |
| History of readmission/ transfer | 3.51 (0.74 – 16.62) | 0.50 (0.06-3.83) |  |
| Prematurity |  |  | <0.001 |
| Full term | Ref | Ref |  |
| Moderate-late preterm | 2.01 (0.18 – 22.41) | 0.75 (0.16-3.42) |  |
| Very preterm | omitted | 1.59 (0.43-5.73) |  |
| Extremely preterm | 21.46* (2.69 – 170.75) | 13.95*** (4.70-41.39) |  |
| Admission diagnosis |  |  | 0.231 |
| Other | Ref | Ref |  |
| RDS | 0.86 (0.22 – 3.38) | 1.38 (0.62-3.10) |  |
| IUGR | 3.34 (0.64 – 17.18) | 0.41 (0.05-3.43) |  |
| Use of antibiotics - Gentamycin |  |  | 0.823 |
| No | Ref | Ref |  |
| Yes | 0.68 (0.20 – 2.26) | 1.01 (0.47-2.19) |  |
| Use of antibiotics - Ampicillin |  |  | 0.144 |
| No | Ref | Ref |  |
| Yes | 6.31 (0.81 – 48.95) | 0.75 (0.39-1.46) |  |
| Use of antibiotics – Piperacillin/tazobactam |  |  | 0.764 |
| No | Ref | Ref |  |
| Yes | 0.92 (0.30 – 2.86) | 1.26 (0.66-2.43) |  |
| Use of antibiotics - Amikacin |  |  | 0.006 |
| No | Ref | Ref |  |
| Yes | 5.70* (1.83 – 17.76) | 0.65 (0.26-1.58) |  |
| Use of antibiotics - Cefotaxime |  |  | 0.250 |
| No | Ref | Ref |  |
| Yes | 2.64 (0.79 – 8.77) | 1.29 (0.55-3.04) |  |
| Use of antibiotics - Meropenem |  |  | 0.001 |
| No | Ref | Ref |  |
| Yes | 8.14*** (2.60 – 25.45) | 0.92 (0.37-2.27) |  |
| Use of antibiotics - Teicoplanin |  |  | 0.001 |
| No | Ref | Ref |  |
| Yes | 7.72* (2.41 – 24.69) | 0.32 (0.04-2.43) |  |
| Use of antibiotics - Vancomycin |  |  | 0.653 |
| No | Ref | Ref |  |
| Yes | 1.67 (0.21 – 13.36) | 1.67 (0.48-5.79) |  |
| Room Number |  |  | 0.529 |
| 1 | Ref | Ref |  |
| 2 | 0.58 (0.10 – 3.21) | 0.77 (0.31-1.94) |  |
| 3 | 1.07 (0.26 – 4.35) | 1.16 (0.51-2.61) |  |
| 4 | 2.79 (0.60 – 12.92) | 1.86 (0.66-5.22) |  |
| Delivery |  |  | 0.722 |
| SVD | Ref | Ref |  |
| Elective C-section | omitted | 0.76 (0.09-6.14) |  |
| Emergency C-section | 1.93 (0.52 – 7.11) | 0.89 (0.45-1.74) |  |
| Other MDROs |  |  | 0.607 |
| No | Ref | Ref |  |
| Yes | 1.15 (0.14-9.09) | 0.36 (0.05-2.72) |  |

* P value <0.05. ** P value <0.01. *** P value <0.001

Supplemental Table 4: Multivariable multinomial logistic regression analysis associating ESBL-KP acquisition and neonatal status at NICU discharge (n=600). The reference group is neonates who survived till NICU discharge.

|  | ***Died- sepsis*** | ***Died -other*** |
| --- | --- | --- |
| **Covariate** | **OR (95%CI)** | **OR (95%CI)** |
| ESBL |  |  |
| No | Ref | Ref |
| Yes | 12.83*(2.41 - 68.22) | 0.24 (0.05-1.09) |
| LOS |  |  |
|  | 0.98 (0.96 – 1.02) | 0.96* (0.94 – 0.98) |
| Use of antibiotics - Amikacin |  |  |
| No | Ref | Ref |
| Yes | 1.17 (0.17 – 7.82) | 1.25 (0.41-3.79) |
| Use of antibiotics - Meropenem |  |  |
| No | Ref | Ref |
| Yes | 6.78 (1.00 – 45.16) | 2.25 (0.70-7.21) |
| Use of antibiotics - Teicoplanin |  |  |
| No | Ref | Ref |
| Yes | 3.80 (0.90 – 16.05) | 1.44 (0.14-14.27) |
| Other MDROs |  |  |
| No | Ref | Ref |
| Yes | 0.69 (0.07-6.58) | 0.75 (0.08-6.31) |

.

* P value <0.05. ** P value <0.01. *** P value <0.001

Supplemental Table 5. Chi-square analysis associating risk factors with ESBL-KP positive cases (BSI vs. colonization). *n*=177

| **Covariate** | ESBL-KP – blood  **Prevalence**  **n (%)** | ESBL-KP- colonization*  **Prevalence**  **n (%)** | ***p*-value** |
| --- | --- | --- | --- |
| Gender |  |  | 0.701 |
| Male | 14 (64) | 92 (59) |  |
| Female | 8 (36) | 63 (41) |  |
| Room Number |  |  | 0.011 |
| 1 | 5 (23) | 60 (39) |  |
| 2 | 4 (18) | 41 (26) |  |
| 3 | 7 (32) | 44 (28) |  |
| 4 | 6 (27) | 10 (6) |  |
| Prematurity |  |  | 0.005 |
| Term | 1 (4) | 15 (10) |  |
| Moderate- Late-term | 3 (14) | 37 (24) |  |
| Very-preterm (28 week - <32 weeks) | 4 (18) | 63 (40) |  |
| Ex-preterm (< 28 weeks) | 14 (64) | 40 (26) |  |
| Mode of Delivery |  |  | 0.787 |
| SVD | 7 (32) | 45 (29) |  |
| Elective C-section | 0 (0) | 3 (2) |  |
| Emergency C-section | 15 (68) | 107 (69) |  |
| Admission diagnosis |  |  | 0.405 |
| Other | 5 (23) | 19 (12) |  |
| RDS | 15 (68) | 119 (77) |  |
| IUGR | 2 (9) | 17 (11) |  |
| MDROs – Other than ESBL-KP |  |  | 0.565 |
| No | 19 (86) | 140 (90) |  |
| Yes | 3 (14) | 15 (10) |  |
| Admission status |  |  | 0.422 |
| New admission | 19 (86) | 142 (92) |  |
| History of readmission/ transfer | 3 (14) | 13 (8) |  |
| Antibiotics - Ampicillin |  |  | 0.168 |
| No | 6 (27) | 24 (15) |  |
| Yes | 16 (73) | 131 (85) |  |
| Antibiotics - Amikacin |  |  | 0.001 |
| No | 7 (32) | 104 (67) |  |
| Yes | 15 (68) | 51 (33) |  |
| Use of antibiotics - Gentamycin |  |  | 0.676 |
| No | 6 (27) | 36 (23) |  |
| Yes | 16 (73) | 119 (77) |  |
| Use of antibiotics - Tazocin |  |  | 0.364 |
| No | 12 (55) | 100 (65) |  |
| Yes | 10 (45) | 55 (35) |  |
| Use of antibiotics - Cefotaxime |  |  | 0.492 |
| No | 17 (77) | 129 (83) |  |
| Yes | 5 (23) | 31 (17) |  |
| Use of antibiotics - Meropenem |  |  | <0.001 |
| No | 7 (32) | 126 (81) |  |
| Yes | 15 (68) | 29 (18) |  |
| Use of antibiotics - Teicoplanin |  |  | <0.001 |
| No | 11 (50) | 136 (88) |  |
| Yes | 11 (50) | 19 (12) |  |
| Use of antibiotics - Vancomycin |  |  | 0.037 |
| No | 18 (82) | 146 (94) |  |
| Yes | 4 (18) | 9 (6) |  |

Supplemental Figure 1: Distribution of Healthcare Associated Infections (HAIs) among the studied sample neonates.


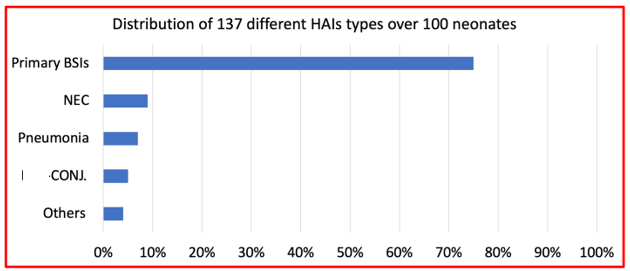


Supplemental Figure 2: Distribution of pathogens isolated from Healthcare Associated Infections (HAIs) among the studied sample neonates.


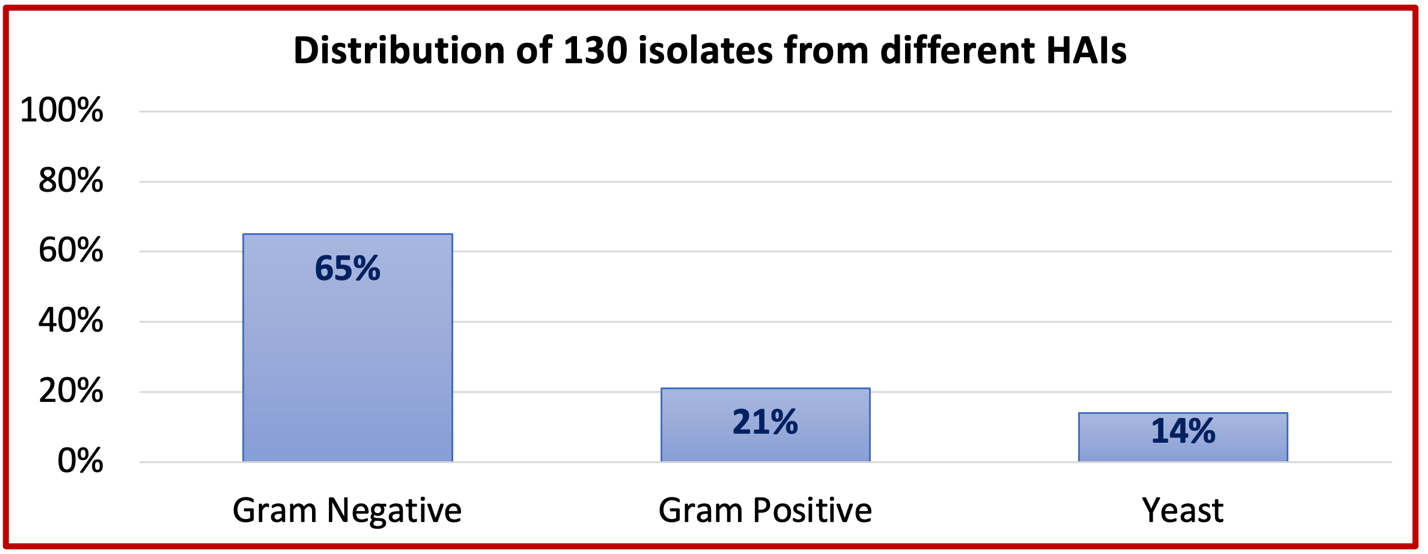


Supplemental Figure 3. Distribution of ESBL-KP among the studied neonate cases.


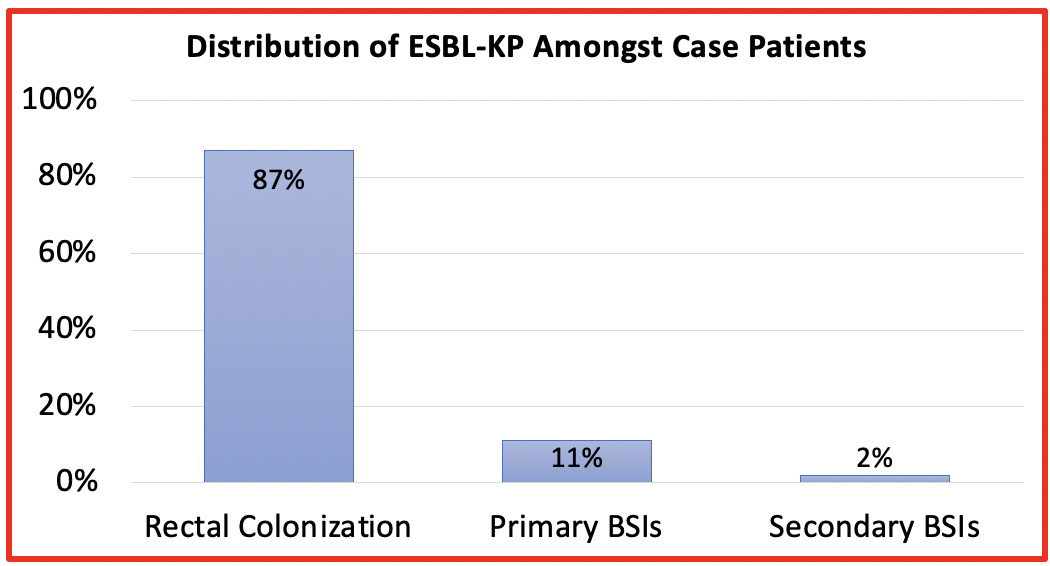

Supplement: Al Ali et al. supplementary material [file S2732494X26107852sup001.docx]
